# Effect of Product Presentation Videos on Consumers' Purchase Intention: The Role of Perceived Diagnosticity, Mental Imagery, and Product Rating

**DOI:** 10.3389/fpsyg.2022.812579

**Published:** 2022-02-17

**Authors:** Zhendong Cheng, Bingjia Shao, Yong Zhang

**Affiliations:** ^1^School of Economics and Business Administration, Chongqing University, Chongqing, China; ^2^Chongqing Key Laboratory of Logistics, School of Economics and Business Administration, Chongqing University, Chongqing, China

**Keywords:** electronic commerce, product presentation videos, perceived diagnosticity, mental imagery, product rating, purchase intention

## Abstract

The product presentation videos on E-commerce platforms have a significant influence on consumers' purchase decisions, and enterprises have focused on choosing the type of product presentation videos. Based on the resource matching theory, mental imagery theory and cue utilization theory, this study investigated the influence of product presentation videos type (product appearance video vs. product usage video) on consumers' purchase intention and the moderating effect of product rating (low vs. high). Through three pre-experiments and two formal experiments, the results showed that the product usage video has a stronger effect on consumers' purchase intention more than the product appearance video, which is mediated by perceived diagnosticity and mental imagery. In addition, product rating moderated the influence of product presentation videos type on consumers' purchase intention. The product usage video would improve consumers' purchase intention more than the product appearance video when the product rating is high; however, there is no significant difference in the impact of two types of videos on consumers' purchase intention when the product rating is low. This study supplements the research on product presentation videos and provides a reference for online retailers to select effective product presentation videos.

## Introduction

With the rapid development of E-commerce, people are increasingly choosing to shop online. According to the report released by E-marketer (2021), it is estimated that the global E-commerce sales will reach $4.92 trillion in 2021, and its proportion in the total retail sales will rise to 19.6%. However, unlike buying products in physical stores, consumers cannot directly view the products, nor can they touch, taste, or try the products when shopping online. So, they rely, to a large extent, on the product presentation information provided by online retailers to judge the product quality and product fit (Jiang and Benbasat, 2004). If consumers cannot obtain enough and needed product information, they will refuse to buy the product (De et al., 2013). Therefore, the major E-commerce platforms and online retailers have focused on optimizing product presentation to convey the product information to consumers more effectively. Now, online merchants are increasingly using videos in addition to traditional image text to present products on the home pages of E-commerce platforms. Videos cannot only provide dynamic visual information and auditory information (Jiang and Benbasat, 2007; Vonkeman et al., 2017) but also, are more vivid than pictures and texts. Furthermore, videos are more suitable for fragmented time to browse. The product experience that videos bring to consumers is closer to the direct product experience in physical stores (Kumar and Tan, 2015). According to a report released by the JD Research Institute (2021) in China, more and more consumers are accustomed to understanding, recognizing, using, and giving feedback on products through product presentation videos. What is more, videos play an important role in consumers' purchase decisions, and high-quality product videos significantly improve the product conversion rates. Although online retailers have devoted an amount of time and money to understanding and creating effective product presentation videos, less is known about which presentation tactics are optimal. Taking canvas shoes as an example, some retailers use videos to mainly present the appearance information of canvas shoes, such as the size, color, and style of canvas shoes, while other retailers use videos to mainly present the user experience information of canvas shoes, such as the overall result of wearing canvas shoes. Which type of product presentation videos improves consumers' purchase intention more effectively? This is an important issue that online retailers face.

However, existing research on product presentation videos is not enough. Prior studies mainly investigated the influence of product presentation videos on product sales (Kumar and Tan, 2015), product attitude (Flavián et al., 2017; Orús et al., 2017), purchase channel selection (Flavián et al., 2017), and purchase intention (Flavián et al., 2017; Orús et al., 2017). In general, previous studies did not classify product presentation videos based on information content. The primary function of product presentation videos is to convey product information to consumers and whether the product information in videos meets consumers' needs will determine the effect of videos (Flavián et al., 2017). Therefore, it is more valuable and necessary to classify videos based on the product information in videos, and then, explore the influence of product presentation videos type on consumers' purchase intention. In addition, prior studies that mainly focused on product factors and consumer factors, proved that product type (Li and Meshkova, 2013; Huang et al., 2017), need for touch (Flavián et al., 2017), information processing motivation (Orús et al., 2017), and impulse buying tendency (Adelaar et al., 2003) are important moderating factors. No scholar has ever investigated the moderating effect of product rating. However, when consumers buy products online, they not only watch the product presentation videos provided by sellers but also view the product rating generated by post-purchase consumers (Utz et al., 2012). The product rating significantly affects the consumers' perception of the truthfulness of product presentation videos. Therefore, investigating the moderating effect of product rating is not only lost to the online shopping situation but also is very valuable in theory.

Based on the above, this study focuses on the following research questions: First, will different types of product presentation videos have different effects on consumers' purchase intention? Second, do perceived diagnosticity and mental imagery mediate the effects? Third, did product rating moderate the effects? To answer the above questions, this study develops a research model to investigate the impact of product presentation videos type on consumers' purchase intention and explore the moderating effect of product rating based on the resource matching theory, mental imagery theory, and cue utilization theory.

## Literature Review and Research Hypotheses

### Online Product Presentation Videos

Scholars defined the online product presentation videos mainly based on their function. For example, Flavián et al. (2017) defined online product presentation videos as audiovisual resources that help online consumers to know the product. Similarly, Orús et al. (2017) proposed that online product presentation videos are audiovisual content showing product characteristics and are used to introduce products to consumers.

Existing research on online product presentation videos can be divided into three categories. Studies in the first category mainly compared product presentation videos with text descriptions (Aljukhadar and Senecal, 2017), pictures (Park et al., 2008; Roggeveen et al., 2015; Huang et al., 2017; Wu et al., 2020; Jai et al., 2021), interactive images (Overmars and Poels, 2015), and virtual experience (Jiang and Benbasat, 2007; Kang et al., 2020; Cowan et al., 2021). These studies investigated the difference between the influence of product presentation videos and other presentation formats on the consumers' cognition, emotion, and behavior intention. Among them, most studies compared product presentation videos with product pictures. The majority of scholars have proposed and proved that product presentation videos are more effective than product pictures. For example, Park et al. (2008) found that product presentation videos (compared with product pictures) increase consumers' perceived information amount, which in turn, would have a significantly positive influence on consumers' emotions. Roggeveen et al. (2015) argued that product presentation videos increase involvement with the product experience in a manner presumably similar to that of the actual product experience and improve consumers' mental imagery. However, some scholars pointed out that product videos are not necessarily better than product pictures; using product pictures (rather than product videos) to present search products will improve product evaluation (Huang et al., 2017).

Studies in the second category focused on product presentation videos and have examined the effect of product presentation videos on product sales (Kumar and Tan, 2015), product attitude (Flavián et al., 2017), purchase intention (Orús et al., 2017), and purchase channel selection (Flavián et al., 2017). For example, Orús et al. (2017) divided product presentation videos into factual videos and animated videos based on the expressing form of videos. They proved that factual videos have a stronger influence on product attitude and purchase intention. In addition, Kumar and Tan (2015) explored the effect of joint product videos on product sales and found that the sales of clothing and accessories significantly increase after online retailers provide product presentation videos.

Studies in the third category combined product presentation videos with other presentation formats, then investigated the influence of different combinations on product preference (Jovic et al., 2012) and consumer trust (Yue et al., 2017). For example, Jovic et al. (2012) combined seven product presentation elements (i.e., text, picture, video, animation, voice, background music, and special effects sound) into nine product presentation formats. The results showed that using both video and image-text presentations have a stronger influence on consumer preference, and the best combination format is text + picture + video + voice + background music. In addition, Yue et al. (2017) named the combination of static pictures + video + 3D image as high media richness presentation. They found that high media richness presentation (vs. picture presentation) significantly reduces perceived risk, which in turn, improved consumer trust.

In general, most of the previous studies have focused on comparing or combining product presentation videos with other presentation formats. There is no research to classify product presentation videos based on information content, and then explore the influence of product presentation videos type on consumers' purchase intention. Furthermore, prior studies mainly focused on product factors and consumer factors proved that product type (Li and Meshkova, 2013; Huang et al., 2017), need for touch (Flavián et al., 2017), information processing motivation (Orús et al., 2017), and impulse buying tendency (Adelaar et al., 2003) are important moderating factors. No scholars have ever investigated the moderating effect of product rating.

### Effect of Product Presentation Videos Type on Consumers' Purchase Intention

According to the definition, the primary function of product presentation videos is to convey product information to consumers, and the information in videos determines the effects of videos largely (Flavián et al., 2017). Therefore, this research focuses on the product information in videos. Based on the main product information provided by videos in current marketing practice and referring to the classification of product review information (Huang et al., 2014; Li et al., 2017), this study divides the product presentation videos into product appearance video and product usage video. The product appearance video mainly contains the product appearance attribute information, such as color, shape, size, and style, while the product usage videos mainly contain the product use experience information, such as the demonstration of product function and the results of product use.

To reveal the influence of product presentation videos on consumers' purchase intention more deeply, this study considers the videos for both search products and experience products. Whether consumers buy search products or experience products online, they tend to make decisions through a two-stage process because they cannot view all the products. In the first stage, that is, the early stage of decision-making process, consumers need to select products that will be further viewed from a large number of products on the list page. In the second stage, that is, the later stage of decision-making process, consumers will deeply evaluate the products that they chose before (Haübl and Trifts, 2000). At the later stage of decision-making process (evaluation stage), consumers tend to use complex and more cognitively effortful decision rules (Weathers et al., 2015). In this stage, consumers want to obtain information about product use experience (Weathers et al., 2015; Li et al., 2017), such as product use method/step, product use process, and product use result. In addition, according to resource matching theory, the balance and match between cognitive resource used to process information and cognitive resource required to process information significantly improve individual judgment and evaluation (Anand and Sternthal, 1989). Information processing is the most efficient and effective when the amount of cognitive resource available matches the amount of cognitive resource required (Mantel and Kellaris, 2003). On the contrary, if the amount of cognitive resource required is more or less than the amount of cognitive resource available, the decision performance of individuals will be poor (Anand and Sternthal, 1989). When the information supplied matches the information demanded, the task performance of individuals will be significantly improved (Anand and Sternthal, 1989). Smith et al. (2011) also proposed that the effectiveness of product presentation is determined by the fit between the information provided and the information sought by consumers. If the information provided by online merchants meets the consumers' need, the consumers' perception of the usefulness of product presentation will be improved, which will help consumers evaluate the product.

In the context of this research, whether consumers purchase search products or experience products on the E-commerce platforms, they will evaluate the product deeply when they enter the product home page through “click a product” on the list. At this time, consumers are in the later stage of the decision-making process and tend to use complex and more cognitively effortful decision rules. They want to obtain product use experience information to make evaluations and purchase decisions (Weathers et al., 2015; Li et al., 2017). Product usage video (vs. product appearance video) provides consumers with more complex product experience information, which requires consumers to make more cognitive efforts. The cognitive resource required to process video information matches the cognitive resource that consumers provide, thus enhancing the persuasiveness of video (Anand and Sternthal, 1989; Meyerslevy and Peracchio, 1995; Peracchio and Meyerslevy, 1997; Hahn and Hwang, 1999). In addition, the product usage video conveys product experience information to consumers, which will satisfy the consumers' information need and stimulate consumers to imagine the use experience (e.g., use process and use effect, etc.), so the consumers' purchase intention will be improved. Therefore, the following hypothesis is proposed:

**Hypothesis 1:** Different types of product presentation videos will have different influences on consumers' purchase intention. The product usage video improves consumers' purchase intention more than the product appearance video.

### Mediating Effect of Perceived Diagnosticity

Perceived diagnosticity refers to the degree to which consumers think that the shopping experience is helpful to evaluate products and make reasonable decisions (Kempf and Smith, 1998). Kempf and Smith (1998) proved that perceived diagnosticity is helpful for consumers to cognitively evaluate product attributes. Jiang and Benbasat (2007) defined perceived diagnosticity as the degree to which consumers think that a website helps them understand products. They found that product presentation videos and virtual product experience improve consumers' perceived diagnosticity, which in turn, has a significantly positive influence on the perceived website usefulness, resulting in a higher intention to return. If consumers feel that they know better about a product, they are more likely to buy it (Berger et al., 1994; Kempf, 1999). In addition, Verhagen et al. (2016) found the positive influence of perceived diagnosticity on consumers' purchase intention in the context of online product presentation.

According to Weathers et al. (2015) and Li et al. (2017), when consumers are in the later stage of decision-making process, they want to get information about product experience, such as product use process, product use method, and product use result. In the context of this research, when consumers enter the product home page through “click a product” on the list, they are in the later stage of decision-making process. Product experience information is exactly what they need, which can help them better understand and be familiar with products. Therefore, the information in product usage video (vs. product appearance video) meets the consumers' need, which can help them understand products, thus improving consumers' perceived diagnosticity. With the perceived diagnosticity increasing, consumers are more likely to buy the products (Verhagen et al., 2016). Thus, the following hypothesis is made:

**Hypothesis 2:** Perceived diagnosticity mediates the effect of product presentation videos type on consumers' purchase intention.

### Mediating Effect of Mental Imagery

As a kind of mental activity that visualizes a concept or relationship (Lutz and Lutz, 1978), mental imagery reflects the process by which individuals represent sensory or perceptual experience, such as thoughts, emotions, and memories in individuals' memory processing (MacInnis and Price, 1987). Mental imagery, as an important theory in consumer psychology, has received extensive attention in consumer behavior studies. In the field of marketing and consumer behavior, the research-related mental imagery theory includes mental imagery and information processing (Edell and Staelin, 1983; MacInnis and Price, 1987), mental imagery and advertising effectiveness (Babin and Burns, 1997; Fennis et al., 2012), mental imagery, and online product presentation (Kim and Lennon, 2008; Yoo and Kim, 2014). According to mental imagery theory, individuals mentally represent stimuli and actions based on what they have experienced in the past, combined with perceptual information available at that moment (Lee and Gretzel, 2012). Consumers can bring the sensory information in memory to their minds through mental imagery awakened by external stimuli, which are being considered as a quasi-sensory experience (Rodríguez-Ardura and Martínez-López, 2014). In addition, consumers can imagine the situations that they have not experienced before and will happen in the future through mental imagery (Schacter et al., 2008), that is, simulating the user experience psychologically. For example, consumers can simulate the situations in resorts (Walters et al., 2007), or imagine the comfort of wearing new sneakers through mental imagery (White et al., 1977). Existing empirical studies have proved that text and visual information in broadcast advertisements, print advertisements, travel advertisements, and online product pictures evoke consumers' mental imagery, which has an impact on consumers' evaluation (such as liking) and consumers' behavior (such as purchase intention) (Bone and Allen, 1992; Babin and Burns, 1997; Walters et al., 2007; Yoo and Kim, 2014; Maier and Dost, 2018). For instance, Burns et al. (1993) argued that the specific words in advertisements will evoke consumers' mental imagery, and then improving consumers' purchase intention. Moreover, Yoo and Kim (2014) found that product pictures with a relevant consumption background are more effective in evoking mental imagery, which in turn, will increase consumers' purchase intention by eliciting a positive emotion.

In the context of this research, the content of product usage video is mainly the demonstration of product function and the results of product use. Compared with product appearance video, the product usage video enables consumers to mentally imagine and simulate the product experience, thus evoking consumers' mental imagery so that they will have a mental simulation experience similar to the actual product experience through mental imagery, resulting in higher purchase intention (Yoo and Kim, 2014; Maier and Dost, 2018). Therefore, the following hypothesis is proposed:

**Hypothesis 3:** Mental imagery mediates the effect of product presentation videos' type on consumers' purchase intention.

### Moderating Effect of Product Rating

Product rating, also called review rating or star rating, is an important form of electronic word of mouth. It is an overall evaluation of products or services in the form of star rating scored by post-purchase consumers (Guo and Zhou, 2016). Most online consumers will search information before making purchase decisions. They not only search for product information provided by sellers but also search for review information generated by post-purchase consumers (Utz et al., 2012). Judging the credibility of sellers' information through electronic word of mouth (such as product star rating) has become the dominant strategy for most consumers (Metzger et al., 2010). Studies have shown that positive electronic word of mouth increases product sales, and negative electronic word of mouth decreases product sales (Chevalier and Mayzlin, 2006; Dellarocas et al., 2007). In addition, electronic word of mouth has a significant impact on consumers' perceived trust in sellers. The more electronic word of mouth is positive, the higher consumers' perceived trust in sellers (Utz et al., 2012).

According to the clue utilization theory, the stability and credibility of product star rating make it more diagnostic. Sellers need to invest a lot of resource to manipulate it, so it belongs to high-scope clues (Purohit and Srivastava, 2001). But the product presentation video is provided by sellers, which is easier to be manipulated than product star rating. In addition, sellers do not need to invest a lot of resource to manipulate product presentation video, so it belongs to low-scope clues. The influence of low-scope clues will be enhanced or weakened by high-scope clues (Purohit and Srivastava, 2001; Miyazaki et al., 2005; Akdeniz et al., 2013). When faced with multiple clues, individuals rely more on high-scope clues to make decisions, and the influence of other clues will be weakened (Hu et al., 2010). Moreover, when faced with consumer review information, the influence of other low-scope clues on consumers will become insignificant (Utz et al., 2012). In the context of this research, when the product rating is high, consumers will trust in sellers, and they think that the product information in videos is true. In addition, the information in product usage video meets the consumers' need and evokes consumers' mental imagery, so the product usage video will improve consumers' purchase intention more than the appearance video. But when the product rating is low, consumers will not trust in sellers and think that the product information in videos is fictitious. Therefore, whether sellers provide the product appearance video or the product usage video for consumers, the purchase intention will be low, and there is no significant difference. Thus, the following hypothesis is formulated:

**Hypothesis 4:** Product rating moderates the effect of product presentation videos' type on consumers' purchase intention. When the product rating is high, the product usage video improves consumers' purchase intention more than the product appearance video, but when the product rating is low, there is no significant difference between the influence of two types of videos on consumers' purchase intention.

**Figure 1:**
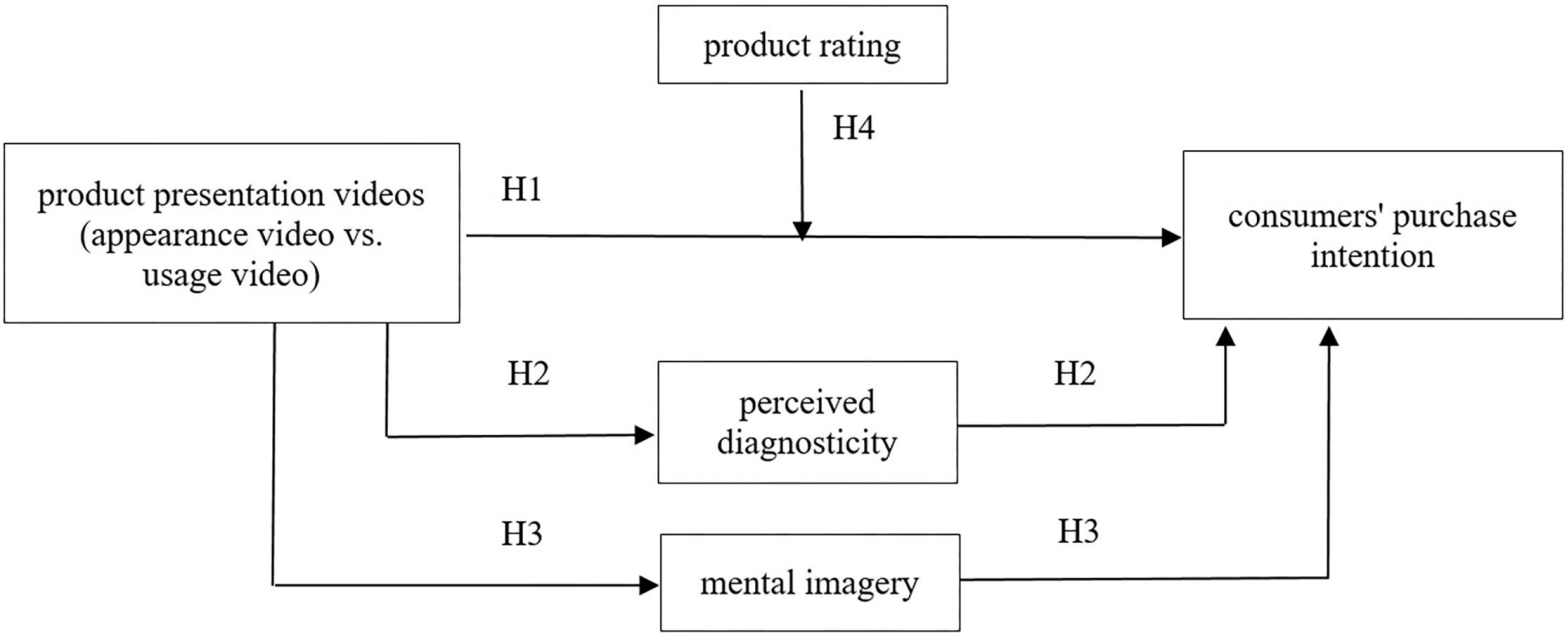
A proposed research model.

The theoretical model is shown in Figure 1.

## Study 1

Study 1 aimed to provide support for H1 that product usage video improves consumers' purchase intention more than product appearance video and for H2, that perceived diagnosticity mediates the effect of product presentation videos' type on consumers' purchase intention, and also for H3, that mental imagery mediates the effect of product presentation videos' type on consumers' purchase intention.

### Pre-experiment 1

To ensure that the products in experiments belong to the search products or the experience products considered by participants, it was necessary to select appropriate products through pre-experiment. Therefore, the purpose of Pre-experiment 1 was to select suitable search products and experience products for Pre-experiment 2, Experiment 1, and Experiment 2. According to the procedure of selecting experimental stimuli in previous studies, we invited three doctoral students majoring in marketing to discuss the definition of product types based on their own online shopping experience. Finally, 6 products (expected to be 3 search products and 3 experience products) were selected as candidate products. These 6 products were: power strips, pillows, backpacks, electric kettles, smart bracelets, and canvas shoes. A total of 81 participants were recruited for Pre-experiment 1 through the “Wenjuanxing” platform, which is a professional questionnaire advisory body in China. At first, the participants were told that the purpose of this experiment was to classify products. Then, the participants were asked to fill in demographic information. Finally, the participants were asked to score 6 products respectively (1 = search products, 7 = experience products) based on the definitions of search products and experience products. The lower the score was, the more it indicated that the participant considered this product to be search product. The higher the score was, the more it indicated that the participant considered this product to be an experience product.

The questionnaires of 81 participants (34 males and 47 females) were all valid. The single-samples *t*-test showed that the average scores of 6 products were consistent with expectation. Specifically, the average scores of canvas shoes (*M* = 5.48, *t* = 19.49, *p* < 0.001), backpacks (*M* = 5.44, t = 20.27, *p* < 0.001), and pillows (*M* = 5.29, *t* = 22.23, *p* < 0.001) were higher than the Median 4, indicating that the participants considered them to be experience products. In addition, the average scores of electric kettles (*M* = 3.22, *t* = 19.71, *p* < 0.001), power strips (*M* = 3.34, *t* = 21.48, *p* < 0.001), and smart bracelets (*M* = 3.78, *t* = 24.83, *p* < 0.001) were lower than the Median 4, indicating that the participants considered them to be search products. Among them, the average score of canvas shoes (*M* = 5.48, *t* = 19.49, *p* < 0.001) was higher than that of the other two experience products, and the average score of electric kettles (*M* = 3.22, *t* = 19.71, *p* < 0.001) was lower than that of the other two search products. Therefore, canvas shoes were selected as the experience products, and electric kettles were selected as the search products, which were used for Pre-experiment 2 and formal experiments.

### Pre-experiment 2

The purpose of Pre-experiment 2 was the manipulation check of product presentation videos' type, and to determine whether the two types of product presentation videos could be used for Experiment 1 and Experiment 2. According to the experience products and search products selected through Pre-experiment 1, we filmed the appearance video of electric kettles, the usage video of electric kettles, the appearance video of canvas shoes, and the usage video of canvas shoes. The duration of each video was 20 s. Specifically, the appearance video of electric kettles mainly contained the appearance attribute information, such as color, size, and design. The usage video of electric kettles mainly presented the steps and procedure of using electric kettles to boil water by a model. The appearance video of canvas shoes mainly contained the appearance attribute information, such as color, size, and style. The usage video of canvas shoes mainly showed the results of wearing canvas shoes by models. A total of 95 participants were recruited for Pre-experiment 2 through the “Wenjuanxing” platform. At first, the participants were told that the purpose of this experiment was to classify product presentation videos. Then, the participants were asked to fill in demographic information and the item about whether they had watched product videos online before. Finally, the participants were asked to score 4 videos respectively (1 = product appearance video, 7 = product usage video) based on the definitions of appearance video and usage video. The lower the score was, the more it indicated that the participant considered this video to belong to the product appearance video. The higher the score was, the more it indicated that the participant considered this video to belong to the product usage video.

The questionnaires of 95 participants (41 males and 54 females) were all valid. The manipulation of product presentation videos' type was examined by independent-samples *t*-test. The result revealed that the average score of electric kettles usage video was higher than that of electric kettles' appearance video (M_appearancevideo_ = 2.57, M_usagevideo_ = 5.42, *t* = −22.16, *p* < 0.001). In addition, the average score of canvas shoes usage video was higher than that of canvas shoes' appearance video (M_appearancevideo_ = 2.77, M_usagevideo_ = 5.45, *t* = −20.52, *p* < 0.001). The manipulation of product presentation videos' type was supported. Therefore, the electric kettles' appearance video and electric kettles' usage video were used for Experiment 1. The canvas shoes' appearance video and canvas shoes' usage video were used for Experiment 2.

### The Design of Experiment 1

This study used an experimental method. Single factor (videos' type: appearance video vs. usage video) between-group designs were implemented. Two videos that passed the manipulation check through Pre-experiment 2 were adopted, namely, the electric kettles' appearance video and the electric kettles' usage video. The two videos were the same in other aspects (e.g., duration, music, image quality, background, etc.) except information content. In addition, brand information was removed to eliminate the influence of product brand. Perceived diagnosticity was measured with three items based on the research of Kempf and Smith (1998), Jiang and Benbasat (2004, 2007), and modified to better reflect the context of this research, specifically “This video is helpful for me to evaluate the electric kettle,” “This video is helpful in familiarizing myself with the electric kettle,” “This video is helpful for me to understand the performance of the electric kettle.” Mental imagery was measured with two items based on the research of Maier and Dost (2018), Yoo and Kim (2014), and modified to better reflect the context of this research, specifically “This video helps me imagine the use process of electric kettle in my mind,” “This video helps me visualize a trial of electric kettle.” Purchase intention was measured with three items based on the research of Fu et al. (2018) and Wu et al. (2020), specifically “I expect to purchase the electric kettle,” “I would consider buying the electric kettle,” “The probability that I would buy the electric kettle is high.” All variables were measured using Likert 7-level scales, where 1 means “strongly disagree” and 7 means “strongly agree.”

### The Procedure of Experiment 1

The “Wenjuanxing” platform was used to design experimental questionnaires and generate links. The participants were also recruited through the “Wenjuanxing” platform. The experimental questionnaire consisted of four parts. The first part told the participants that this experiment was about investigating consumers' behavior. The second part described the experimental scenario, specifically “Imagine that you are shopping on an E-commerce platform like Tmall.com. After searching for “electric kettles,” you enter the category overview page. Then, you click an electric kettle on the category overview page to enter the product home page. You see the following product video on the product home page. Please complete the items after watching the video.” The third part was the measurement scales of related variables. In addition, the measurement item about product presentation video types was set to test the manipulation effectiveness of this experiment. The fourth part contained demographic information, such as the gender, education, and age of the participants.

The participants were randomly assigned to one of the two experimental groups (the appearance video group vs. the usage video group). At first, the participants imagined that they were on the product home page of an E-commerce platform by reading descriptions. Then, they would see the product presentation videos corresponding to their experimental groups. Finally, the participants would complete the relevant scales and demographic information. A total of 163 participants participated in the experiment, and 144 participants completed the experiment. The demographic information of participants is shown in Table 1 (Akbari et al., 2019).

**Table 1:** Demographic information of participants in Experiment 1.

**Variables**	**Distribution**	**Numbers**	**%**
Gender	Male	63	43.8
	Fmale	81	56.2
Age	Under 20	50	34.7
	20–25 years old	55	38.2
	26–30 years old	29	20.1
	30 years and older	10	7.0
Education	High school and below	2	1.4
	College students	3	2.1
	Undergraduate	96	66.7
	Postgraduate	43	29.8
Monthly consumption expenditure	Less than 1,000 yuan	36	25.0
	1,000–2,000 yuan	73	50.7
	2,001–3,000 yuan	21	14.6
	More than 3,000 yuan	14	9.7

### The Results of Experiment 1

#### Manipulation Check

The manipulation of product presentation videos' type was examined by independent-samples *t*-test. The result showed that the average score of electric kettles' usage video was higher than that of electric kettles' appearance video (M_appearancevideo_ = 2.23, M_usagevideo_ = 5.91, *t* = −14.29, *p* < 0.01). The manipulation of product presentation videos' type was supported.

#### Main Effect Testing

**Figure 2:**
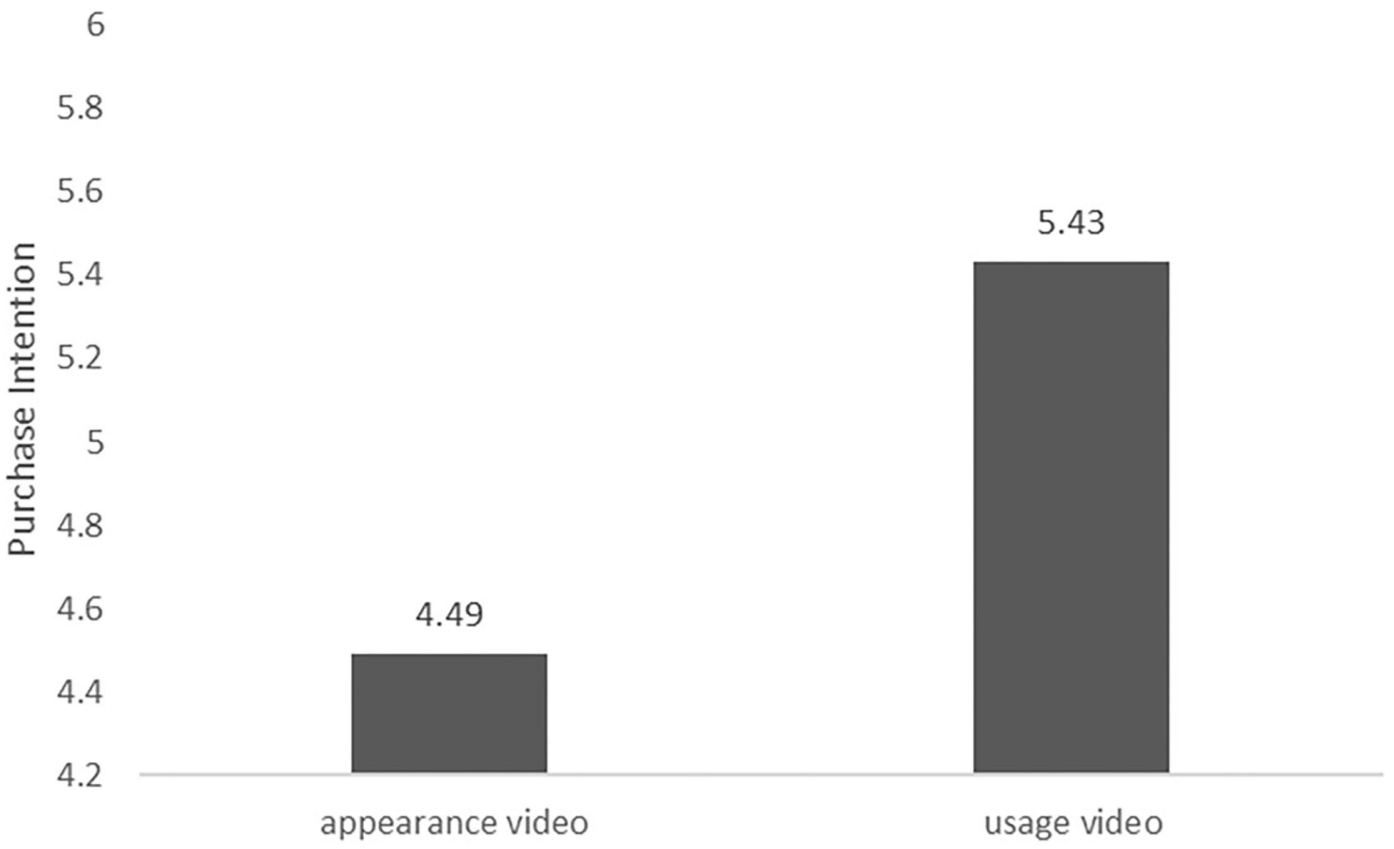
Difference in purchase intention by the appearance video and the usage video (for a search product).

The test was performed using ANOVAs, and the results are shown in Figure 2. The main effect of product presentation videos' type on consumers' purchase intention was significant [*F*_(1, 142)_ = 10.78, *p* < 0.01], where M_appearancevideo_ = 4.49, M_usagevideo_ = 5.43. It was indicated that the product usage video improves consumers' purchase intention more than the product appearance video. Therefore, H1 was verified.

#### Mediating Effect Testing

The bootstrapping method was used to test the mediating effect of perceived diagnosticity and mental imagery (Hayes, 2013). The analytic approach was informed by Preacher et al. (2007) who recommend bias-corrected bootstrapping to measure multiple indirect effects. In this case, 5,000 samples were taken, and the confidence level was selected as 95%. The total indirect effect for two mediators assessed simultaneously was significant (*Z* = 8.61, *p* < 0.01), which is consistent with the hypothesis that perceived diagnosticity and mental imagery mediate the effect of product presentation videos' type on consumers' purchase intention. We then examined the mediators individually, and the result showed that a 95% confidence interval for the indirect path through perceived diagnosticity was significant, β = 0.1069 (LLCI = 0.0339, ULUI = 0.2028, excluded 0), indicating that the mediation effect of perceived diagnosticity between product presentation videos' type and consumers' purchase intention was significant. Therefore, H2 was supported. In addition, a 95% confidence interval for the indirect path through mental imagery was significant, β = 0.2685 (LLCI = 0.1509, ULUI = 0.4137, excluded 0), indicating that the mediation effect of mental imagery between product presentation videos' type and consumers' purchase intention was significant. Therefore, H3 was supported. To determine the relative value of the two mediators, we conducted bias-corrected comparisons between mediators. The 95% confidence intervals for contrasts of perceived diagnosticity with mental imagery did not include zero, indicating that mental imagery was a significantly stronger mediator than perceived diagnosticity.

## Study 2

Study 2 aimed to provide support for H4 that product rating moderates the effect of product presentation videos' type on consumers' purchase intention.

### Pre-experiment 3

The purpose of Pre-experiment 3 was the manipulation check of product rating, and to determine whether the two types of product rating could be used for Experiment 2. After observing the distribution of product rating on major E-commerce platforms and referring to Chu et al. (2015), the low product rating was manipulated into a two-star rating and the high product rating was manipulated into a four-star rating. A total of 58 participants were recruited for Pre-experiment 3 through the “Wenjuanxing” platform. At first, the participants were told that the purpose of this experiment was to judge the product rating. Then, the participants were asked to fill in demographic information and the item about whether they had viewed the product rating online before. Finally, the participants were asked to score the two-star rating and the four-star rating, respectively (1 = low product rating, 7 = high product rating). The lower the score was, the more it indicated that the participant considered this product rating to belong to the low product rating. The higher the score was, the more it indicated that the participant considered this product rating to belong to the high product rating.

The questionnaires of 58 participants (31 males and 27 females) were all valid. The manipulation of product rating was examined by independent-samples *t*-test. The result showed that the average score of the four-star rating was higher than that of the two-star rating (M_two−star_ = 2.63, M_four−star_ = 5.74, *t* = −21.37, *p* < 0.001). The manipulation of the product rating was supported. Therefore, the two-star rating was used as the low product rating, and the four-star product rating was used as high the product rating for Experiment 2.

### The Design of Experiment 2

This study used an experimental method. About 2 (videos' type: appearance video vs. usage video)- × -2 (product rating: low vs. high) between-group designs were implemented. Two videos that passed the manipulation check through Pre-experiment 2 were adopted, namely, canvas shoes' appearance video and canvas shoes' usage video. The two videos were the same in other aspects (e.g., duration, music, image quality, background, etc.) except information content. In addition, brand information was removed to eliminate the influence of product brand. Perceived diagnosticity was measured with three items based on the research of Kempf and Smith (1998), Jiang and Benbasat (2004, 2007), and modified to better reflect the context of this research, specifically “This video is helpful for me to evaluate the canvas shoes,” “This video is helpful in familiarizing myself with the canvas shoes,” “This video is helpful for me to understand the performance of the canvas shoes.” Mental imagery was measured with two items based on the research of Maier and Dost (2018), Yoo and Kim (2014), and modified to better reflect the context of this research, specifically “This video helps me imagine the results of wearing canvas shoes in my mind,” “This video helps me visualize a trial of canvas shoes.” Purchase intention was measured with three items based on the research of Fu et al. (2018) and Wu et al. (2020), specifically “I expect to purchase the canvas shoes,” “I would consider buying the canvas shoes,” “The probability that I would buy the canvas shoes is high.” All variables were measured using Likert 7-level scales, where 1 means “strongly disagree” and 7 means “strongly agree.”

### The Procedure of Experiment 2

The “Wenjuanxing” platform was used to design experimental questionnaires and generate links. The participants were also recruited through the “Wenjuanxing” platform. The experimental questionnaire consisted of five parts. The first part told the participants that this experiment was about investigating consumers' behavior. The second part described the experimental scenario, specifically “Imagine that you are shopping on an E-commerce platform like Tmall.com. After searching for “canvas shoes,” you enter the category overview page. Then, you click canvas shoes on the category overview page to enter the product home page. You see the following product video on the product home page.” The third part was also a description, specifically “After watching the product video, you further view the product rating, and find that the product rating is two/four stars, please complete the flowing items.” The fourth part was the measurement scales of related variables. In addition, the measurement items about product presentation video types and product rating were set to test the manipulation effectiveness of this experiment. The last part contained demographic information, such as the gender, education, and age of the participants.

The participants were randomly assigned to one of the four experimental groups. At first, the participants imagined that they were on the product home page of an E-commerce platform by reading descriptions. Then, they would see the product presentation videos corresponding to their experimental groups. After that, they would be informed of the product rating. Finally, the participants would complete the same scales in Experiment 1 and demographic information. A total of 202 participants participated in the experiment, and 188 participants completed the experiment. The demographic information of the participants is shown in Table 2 (Akbari and Moradipour, 2021).

**Table 2:** Demographic information of the participants in Experiment 2.

**Variables**	**Distribution**	**Numbers**	**%**
Gender	Male	78	41.5
	Fmale	110	58.5
Age	Under 20	71	37.8
	20–25 years old	74	39.3
	26–30 years old	28	14.9
	30 years and older	15	8.0
Education	High school and below	3	1.6
	College students	9	4.8
	Undergraduate	122	64.9
	Postgraduate	54	28.7
Monthly consumption expenditure	Less than 1,000 yuan	73	38.8
	1,000–2,000 yuan	79	42.1
	2,001–3000 yuan	22	11.7
	More than 3000 yuan	14	7.4

### The Results of Experiment 2

#### Manipulation Check

The manipulation of product presentation videos' type was examined by independent-samples *t*-test. The result showed that the average score of canvas shoes' usage video was higher than that of canvas shoes' appearance video (M_appearancevideo_ = 2.26, M_usagevideo_ = 5.83, *t* = −13.37, *p* < 0.01). The manipulation of product presentation videos' type was supported.

The manipulation of the product rating was also examined by independent-samples *t*-test. The result showed that the average score of the four-star rating was higher than that of the two-star rating (M_two−star_ = 2.21, M_four−star_ = 5.61, *t* = −12.28, *p* < 0.01). The manipulation of the product rating was also supported.

#### Main Effect Testing

**Figure 3:**
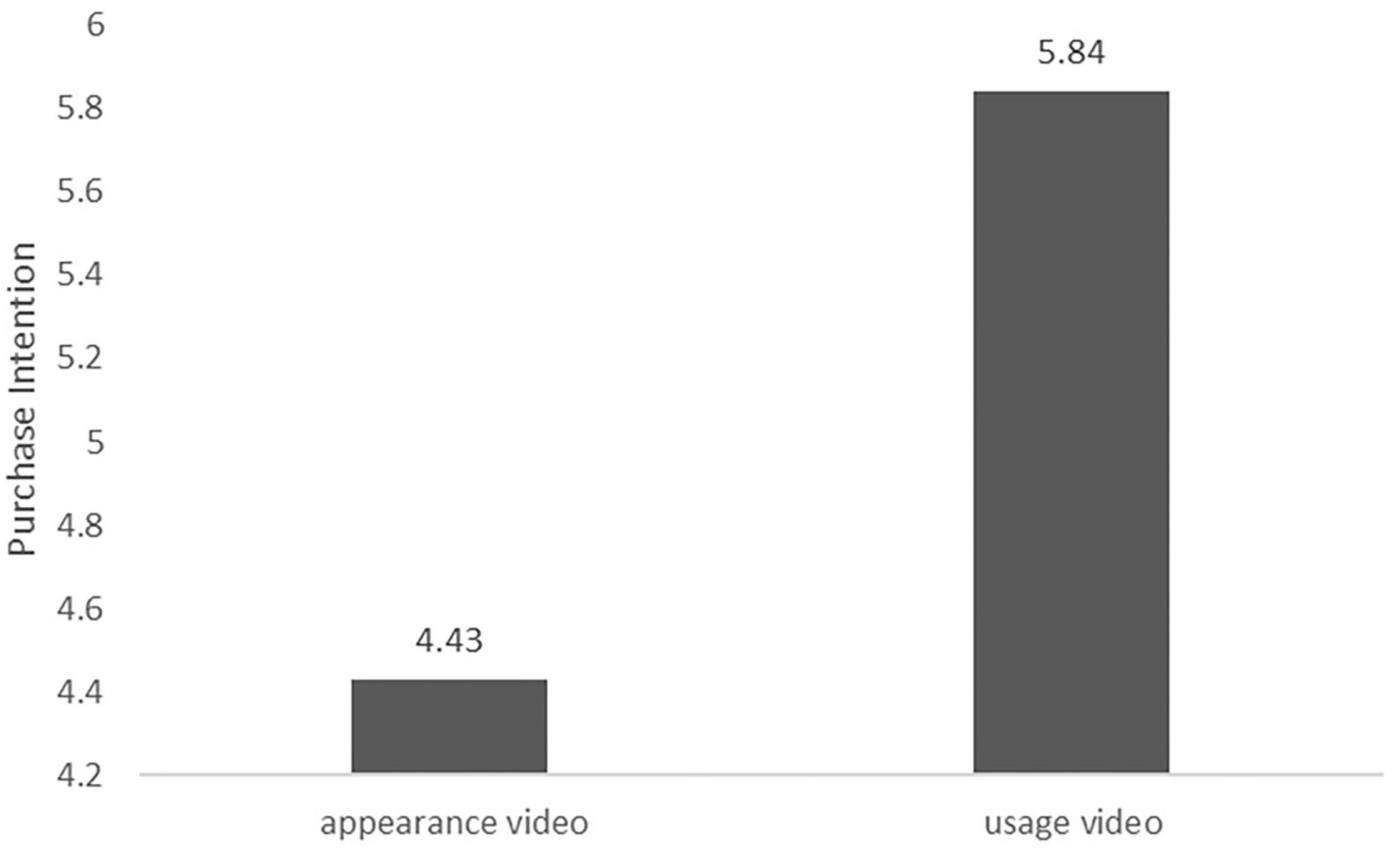
Difference in purchase intention by the appearance video and the usage video (for an experience product).

The test was performed using ANOVAs, and the results are shown in Figure 3. The main effect of product presentation videos' type on consumers' purchase intention was significant [*F*_(1, 186)_ = 13.81, *p* < 0.01], where M_appearancevideo_ = 4.43, M_usagevideo_ = 5.84. It was indicated that product usage videos improve consumers' purchase intention more than product appearance videos. Therefore, H1 was verified again.

#### Mediating Effect Testing

The bootstrapping method was used to test the mediating effect of perceived diagnosticity and mental imagery (Hayes, 2013). In this case, 5,000 samples were taken, and the confidence level was selected as 95%. The total indirect effect for two mediators assessed simultaneously was significant (*Z* = 9.38, *p* < 0.01), which is consistent with the hypothesis that perceived diagnosticity and mental imagery mediate the effect of product presentation videos' type on consumers' purchase intention. We then examined the mediators individually, and the result showed that a 95% confidence interval for the indirect path through perceived diagnosticity was significant, β = 0.1272 (LLCI = 0.0627, ULUI = 0.1832, excluded 0), indicating that the mediation effect of perceived diagnosticity between product presentation videos' type and consumers' purchase intention was significant. Therefore, H2 was supported again. In addition, a 95% confidence interval for the indirect path through mental imagery was significant, β = 0.3047 (LLCI = 0.1923, ULUI = 0.4566, excluded 0), indicating that the mediation effect of mental imagery between product presentation videos' type and consumers' purchase intention was significant. Therefore, H3 was supported again. To determine the relative value of the two mediators, we conducted bias-corrected comparisons between mediators. The 95% confidence intervals for contrasts of perceived diagnosticity with mental imagery did not include zero, indicating that mental imagery was a significantly stronger mediator than perceived diagnosticity.

#### Moderating Effect Testing

**Figure 4:**
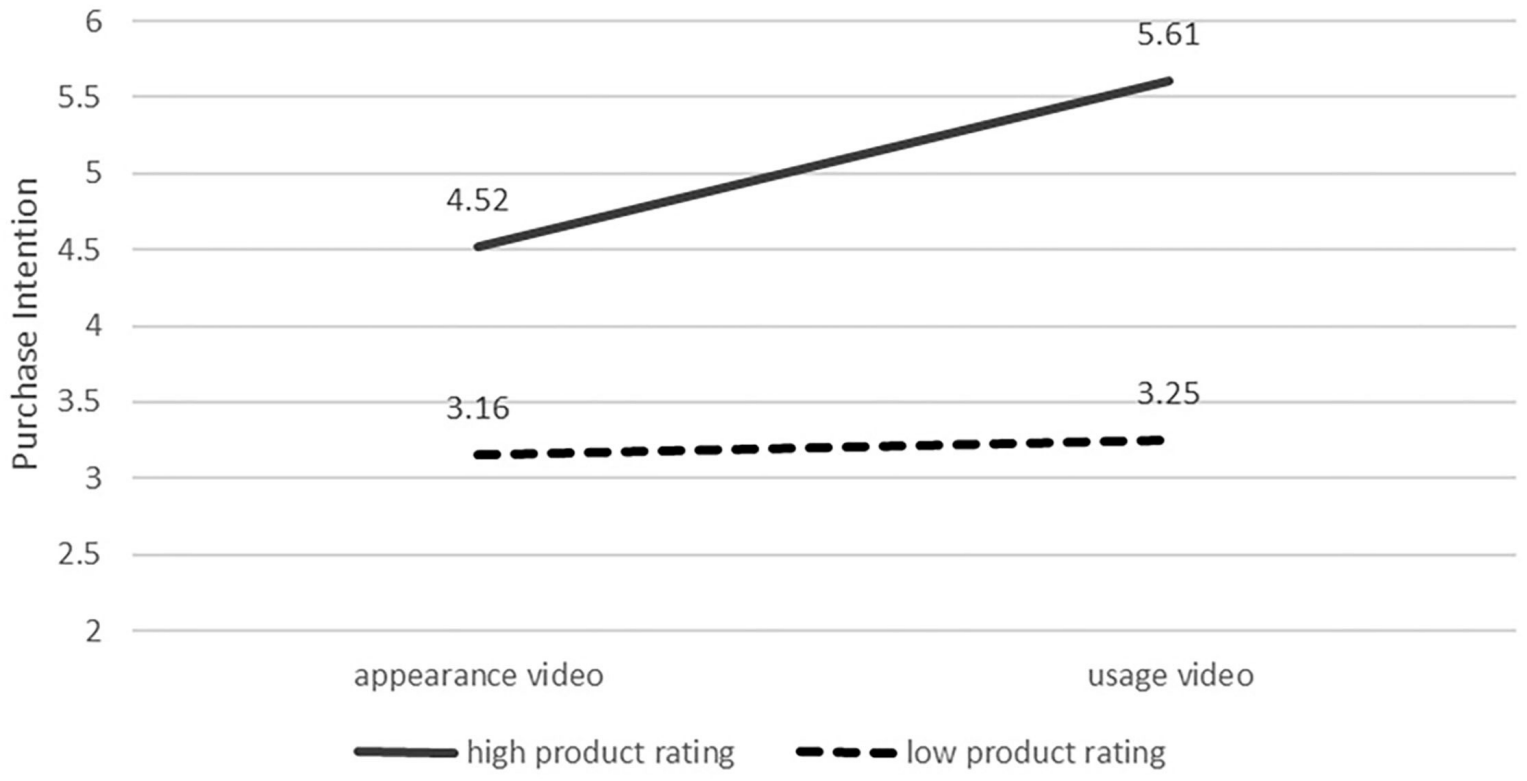
Purchase intention in the different conditions of product rating.

With purchase intention as the dependent variable, videos' type and product rating as the independent variables, a 2 (videos' type: appearance video vs. usage video)- × -2 (product rating: low vs. high) ANOVAs revealed a significant interaction effect of videos' type with the product rating [*F*_(1, 185)_ = 12.62, *p* < 0.01], which indicates that the product rating (low vs. high) moderated the effect of product presentation videos' type on consumers' purchase intention. Furthermore, simple effect analysis showed that the purchase intention of the participants who were assigned to the usage video group (M_usagevideo_ = 5.61) was significantly higher than that of the participants who were assigned to the appearance video group (M_appearancevideo_ = 4.52) in the condition of the high product rating [*F*_(1, 92)_ = 10.11, *p* < 0.01], as shown in Figure 4. There was no significant difference between the appearance video group and the usage video group in the condition of the low product rating [M_appearancevideo_ = 3.16,M_usagevideo_ = 3.25, *F*_(1, 93)_ = 2.01, *p* >0.1]. Therefore, H4 was supported.

## General Discussion

### Summary

Based on the resource matching theory, mental imagery theory, and cue utilization theory, this study investigated the influence of product presentation videos' type (appearance video vs. usage video) on consumers' purchase intention and examined the moderating effect of the product rating (low vs. high). Moreover, the mediating role of perceived diagnosticity and mental imagery was also verified.

There are three findings in this study: First, the product presentation videos' type has a significant effect on consumers' purchase intention. The product usage video improves consumers' purchase intention more than the product appearance video for both search products and experience products. Second, perceived diagnosticity and mental imagery mediate the influence of product presentation videos' type on consumers' purchase intention. The product usage video improves consumers' perceived diagnosticity and mental imagery more than the product appearance video, resulting in higher purchase intention. Third, product rating moderates the influence of product presentation videos' type on consumers' purchase intention. The product usage video improves consumers' purchase intention more than the product appearance video when the product rating is high; however, there is no significant difference in the impact of two types of videos on consumers' purchase intention when the product rating is low.

### Theoretical Contributions

This research has contributed to product presentation videos, product rating, and the resource matching theory. First, this study enriches the classification of product presentation videos. Previous studies mainly classified product presentation videos based on the expressing form of videos, but the information in different videos was kept the same. However, the effect of product presentation videos depends, to a large extent, on the information in videos. Focusing on the information content and referring to the classification of product review information (Huang et al., 2014; Li et al., 2017), this study divides the product presentation videos into product appearance video and product usage video.

Second, this study proposes and validates the moderate effect of the product rating, which is a new boundary condition for the influence of product presentation videos on consumers' purchase intention. Prior studies mainly focused on product factors and consumer factors, proved that product type, need for touch, information processing motivation, and impulse buying tendency are important moderating factors; no studies have ever investigated the moderating effect of product rating. However, when consumers buy products online, they not only watch the product presentation videos provided by sellers but also view the product rating generated by post-purchase consumers (Utz et al., 2012). The product rating significantly affects the consumers' perception of the truthfulness of product presentation videos.

Third, this study extends the application of resource matching theory by introducing it into the research field of online product presentation videos. Past studies mainly based on the dual coding theory and the media richness theory to explore the influence of product presentation videos on consumers' behavior. Focusing on matching between cognitive resource needs and supplies, this study finds that providing product usage video for consumers on the product home pages will make consumers' cognitive resource needs match the cognitive resource supplies, resulting in higher purchase intention.

### Implications

The current research is of great significance to the marketing practice of enterprises. First, this study would suggest online merchants pay attention to the selection and management of product presentation videos. Consumers cannot directly experience the products when shopping online, so they have higher perceived uncertainty and perceived risk. If consumers cannot obtain enough and needed product information, they will refuse to buy the product. Therefore, it is very important for online merchants to design and manage product presentation videos effectively. This study finds that product presentation videos' type influences consumers' purchase intention significantly, which provides reasons for online merchants to attach importance to the management of product presentation videos.

Second, this study provides a reference for online merchants to design effective product presentation videos on product home pages. The results of this study show that product usage video improve consumers' purchase intention more than product appearance video for both search products and experience products. So, the online merchants should present the product usage videos to consumers on product home pages. In addition, online merchants should consider the specific characteristics of search products and experience products when designing the product usage videos. Specifically, for search products, the content of videos should be the demonstration of the use procedure and function by models. Taking smart bracelets as an example, it is better to use videos to demonstrate the functions of smart bracelets so as to vividly show consumers how to use the smart bracelets. For experience products, the content of videos should be the demonstration of use results and performance by models. Taking T-shirts as an example, it is better to use videos to show the image of wearing the T-shirt by models so as to let consumers see what it looks like after wearing the T-shirt.

Third, this study is helpful for online merchants to rationally recognize the influence of product presentation videos on consumers' purchase decisions. The results of this study show that product rating moderates the influence of product presentation videos' type on consumers' purchase intention. When the product rating is low, consumers will not trust in sellers and think that the product information in the videos is fictitious. Therefore, on the one hand, online merchants should not blindly exaggerate the product performance through product presentation videos, or else will lower the product rating, and then weaken the effects of product presentation videos. On the other hand, online merchants should not only ensure the information in videos is true and the quality of products is high but also pay attention to guiding consumers to give products higher ratings, which will strengthen the effects of product presentation videos. For example, online merchants could encourage consumers to make higher rating by giving coupons.

### Limitations and Further Research

Whereas, the findings of this study are valid and valuable, there are still some limitations that provide directions for further research. First, the participants of the experiments in this study were mainly students instead of general populations of consumers. The reason is that the homogeneity of students is relatively high and other interference variables are relatively few. In addition, students have rich online shopping experience and are the desired representatives of online shoppers. Future research can expand the scope of the sampling groups to enhance the universality of the research. Second, this study explored the influence of product presentation videos on consumers' purchase intention through scenario experiments. The reason is that this study belongs to the causal exploratory research about consumer behaviors, which requires to control many factors strictly. So, we used the method of the scenario experiment, which can ensure high internal validity. Big data and other quantitative methods can be used for further research, such as cooperating with online merchants and conducting natural experiments to further verify the relationship between product presentation videos' type and product sales. Third, only two kinds of products were selected for analysis in this study. The reason is that the results of Pre-experiment 1 showed that the participants consider electric kettles and canvas shoes are most consistent with the definition of search products and experience products, so the two kinds of products are more representative. In addition, electric kettles and canvas shoes are similar in price, importance, and purchase frequency, which can eliminate the influence of some interference factors, such as price, involvement, and familiarity. Future research could use other products for verification and improve the robustness of research. Lastly, the duration of product videos in experiments is all 20 s, without considering the possible influence of time pressure. In addition, the product presentation videos investigated in this study were shot and provided by merchants, without considering the possible impact of video co-creation. The future study could investigate the influence of videos with different lengths of duration and the influence of videos created by buyers-sellers on purchase intention.

## Data Availability Statement

The raw data supporting the conclusions of this article will be made available by the authors, without undue reservation.

## Ethics Statement

The studies involving human participants were reviewed and approved by Economics and Business Administration Department Research Ethic Committee. The patients/participants provided their written informed consent to participate in this study.

## Author Contributions

ZC contributed to the conceptualization, methodology, statistical analysis, data curation, and writing. BS contributed to the revision, investigation, supervision, funding acquisition, and project administration. YZ contributed to the revision and funding acquisition. All the authors contributed to the manuscript revision and read and approved the submitted version.

## Funding

We acknowledge the financial support from the National Natural Science Foundation of China (grant nos.: 72110107002, 71974021), the Fundamental Research Funds for the Central Universities of Chongqing University, project No. 2019 CDJSK 02 XK 12.

## Conflict of Interest

The authors declare that the research was conducted in the absence of any commercial or financial relationships that could be construed as a potential conflict of interest.

## Publisher's Note

All claims expressed in this article are solely those of the authors and do not necessarily represent those of their affiliated organizations, or those of the publisher, the editors and the reviewers. Any product that may be evaluated in this article, or claim that may be made by its manufacturer, is not guaranteed or endorsed by the publisher.
